# Wednesday Wisdom: Implementing a Bitesize Multidisciplinary Teaching Programme on an Inpatient Psychiatric Ward

**DOI:** 10.1192/bjo.2026.11342

**Published:** 2026-06-30

**Authors:** Lauren Bone, Tariq Mahmood

**Affiliations:** Leeds and York Partnership NHS Foundation Trust, Leeds, United Kingdom

## Abstract

**Aims:**

Teaching on an inpatient acute ward is often ad-hoc and deprioritised due to clinical pressures, with structured teaching usually targeted primarily at medical trainees. The aim of this project was to establish a weekly ‘bitesize’ teaching programme delivered immediately following the Wednesday morning huddle. The objectives were to improve shared understanding of physical health and core psychiatric topics, improve inter-disciplinaryunderstanding and to create a positive culture of regular, expected and structured MDT teaching on the ward.

**Methods:**

A weekly ‘bitesize’ teaching programme called *Wednesday wisdom* was established and delivered immediately following the Wednesday morning team huddle. Sessions were designed to be time-efficient and sustainable within a busy inpatient environment, lasting 10–15 minutes. Members of staff from multiple disciplines were contacted to deliver sessions, including pharmacists, dieticians, chaplaincy and mental health nurses.

Anonymous pre-programme questionnaires were distributed to the MDT to assess perceived adequacy of current teaching opportunities on the ward and acceptability of the proposed programme.

**Results:**

Baseline questionnaires (n=7) highlighted a lack of structured MDT teaching, with 71% of respondents reporting that there were not enough opportunities for MDT teaching on the ward. Mean self-rated confidence scores were 6.4/10. There was a high perceived likelihood of attendance if the sessions were scheduled immediately after the Wednesday morning huddle (72%).

Post-programme feedback demonstrated high satisfaction across all measured domains. 100% of respondents wanted the programme to continue and would recommend the format to other wards. Mean scores were high for relevance to role 9.7/10, being pitched at an appropriate level 9.3/10, appropriate session length 10/10, overall usefulness 9.6/10, increased confidence at applying learning 9.4/10, inclusivity and relevance to MDT 9.5/10 and integration intoward routine 10/10. A small number of respondents suggested refining pharmacy and medically focused sessions to ensure accessibility for the wider MDT.

**Conclusion:**

This programme demonstrates that short and structured MDT teaching embedded within existing ward structures can be a highly effective way of improving learning opportunities in a busy inpatient psychiatric setting and can be delivered with minimal resources.

